# Promising Immunomodulatory Effects of Bacterial Lysates in Allergic Diseases

**DOI:** 10.3389/fimmu.2022.907149

**Published:** 2022-06-22

**Authors:** Agnieszka Kaczynska, Martyna Klosinska, Kamil Janeczek, Michał Zarobkiewicz, Andrzej Emeryk

**Affiliations:** ^1^ Department of Pulmonary Diseases and Children Rheumatology, Medical University of Lublin, Lublin, Poland; ^2^ Department of Clinical Immunology, Medical University of Lublin, Lublin, Poland

**Keywords:** adaptive immunity, bacterial lysate, asthma, allergic rhinitis, atopic dermatitis, innate immunity

## Abstract

In light of an escalating prevalence of allergic disorders, it is crucial to fully comprehend their pathophysiology and etiology. Such knowledge would play a pivotal role in the search for new therapeutic approaches concerning not only diseases’ symptoms, but also their underlying causes. The hygiene hypothesis indicates a high correlation between limited exposure to pathogens in early childhood and the risk of developing allergic disorders. Bearing in mind the significance of respiratory and digestive systems’ mucous membrane’s first-line exposure to pathogens as well as its implications on the host’s immune response, a therapy targeted at aforesaid membranes could guarantee promising and extensive treatment outcomes. Recent years yielded valuable information about bacterial lysates (BLs) known for having immunomodulatory properties. They consist of antigen mixtures obtained through lysis of bacteria which are the most common etiologic agents of respiratory tract infections. They interact with dendritic cells located in the mucous membranes of the upper respiratory tract and the gastrointestinal tract by toll-like receptors. The dendritic cells present acquired antigens resulting in innate immune response development on the release of chemokines, both stimulating monocytes and NK cells maturation and promoting polymorphonuclear neutrophil migration. Moreover, they influence the adaptive immune system by stimulating an increase of specific antibodies against administered bacterial antigens. The significance of BLs includes not only an anti-inflammatory effect on local infections but also restoration of Th1/Th2 balance, as demonstrated mainly in animal models. They decrease Th2-related cytokine levels (IL-4, IL-13) and increase Th1-related cytokine levels (IFN-γ). The reestablishment of the balance of the immune response leads to lowering atopic reactions incidence which, in addition to reduced risk of inflammation, provides the alleviation and improvement of clinical manifestations of allergic disorders. In this review, we hereby describe mechanisms of BLs action, considering their significant immunomodulatory role in innate immunity. The correlation between local, innate, and adaptive immune responses and their impact on the clinical course of allergic disorders are discussed as well. To conclude our review, we present up-to-date literature regarding the outcomes of BLs implemented in atopic dermatitis, allergic rhinitis, and asthma prevention and treatment, especially in children.

## Introduction

Recent decades followed the endurance of tremendous changes in the environment, economic and social developments alongside worldwide urbanization (1). Unfortunately, that led to an increased prevalence of allergic disorders estimated at 20% of the population, especially children (2). Possible collocations are also noticed regarding atopic sensitization and hyperresponsiveness in the upper airway (3). The ‘atopic march’ refers to an observed incidence of atopic dermatitis (AD) and food allergies in infancy, gradually progressing into allergic rhinitis (AR) and allergic asthma later in childhood (4). Asthma development is observed in 30% of children with AD (5). Speculations surrounding atopic diseases center around adaptive immune response disturbances with consideration of Th1/Th2 imbalance as well as the hygiene hypothesis, underlying the crucial role of innate immunity (6). Therapeutic options regard lifestyle changes, such as food interventions and environmental prevention, but also focus on finding an appropriate pharmacological treatment (7–9). Current guidelines recommend multiple medications that alleviate allergy symptoms and improve quality of life. However, treatment has several limitations and is burdened with side effects; thus, introducing new therapeutic approaches remains crucial. Bacterial lysates (BLs) seem to be an effective and safe way of allergy prevention and treatment. They consist of inactivated respiratory tract bacteria obtained by mechanical or chemical lysis. Their significant mechanism of action enables them to modulate the immune response by activating innate immunity and, as the data suggest, by restoring Th1/Th2 balance (10, 11).

This publication aimed to summarize and explain the immunomodulatory effects of BLs in the prevention and treatment of patients (mainly children) with AD, AR, and asthma. Furthermore, the clinical outcomes of this treatment were discussed as well.

This narrative review consists of a presentation and analysis of literature and previously published studies obtained from PubMed, Scopus, and Google Scholar databases. In all the databases, we used the combination of the following keywords: ‘atopic dermatitis’, ‘allergic rhinitis’, ‘asthma’, ‘bacterial lysate’, ‘bacterial extract’, ‘innate immunity’, ‘adaptive immunity’, ‘mechanism of action’, ‘immunomodulation’ and ‘immunomodulatory properties’.

## Immunological, Genetic, and Epigenetic Factors of Allergy Development

During years of evolution, the immune system has developed numerous mechanisms necessary to eliminate harmful environmental factors. Pathogens, allergens, and pollutants are recognized and neutralized every day. Both innate and adaptive immune responses work closely to prevent infections. However, sometimes the reaction becomes too intense. This phenomenon, called hypersensitivity, is a cause of multiple diseases such as allergies, autoimmune disorders, and transplant rejections. This review will focus mainly on the immediate type of hypersensitivity as it is typical for allergies. It is characterized by the increased level of IgE antibodies specific to allergens (asIgE) as well as the disturbance of Th1/Th2 immune response balance, which combined with chronic inflammation in the mucosa leads to burdensome allergic symptoms (12).

Many years of observations indicate numerous factors that affect the risk of allergy development. These include genetic factors, exposure to allergens in childhood, food consumed by the child during early life, infectious and toxic agents (13).

The hygiene hypothesis seems to be the most reliable explanation for the rising allergy incidence. It is closely linked with the decrease of infectious disease burden due to vaccines, antibiotics, and hygiene measures. It has been proven that prenatal or early-life immunostimulatory signals significantly impact the development of the immune system (14, 15). The ‘old friends’ hypothesis points out the beneficial role of symbiotic bacteria and parasites in dendritic cells’ maturation and maintenance of Th1/Th2 balance (16). This hypothesis suggests that a key to treating allergic disorders may be a controlled presentation of antigens that could replace mentioned early-life exposure (17, 18).

Clinical findings show a strong correlation between various genetic factors and the severity and treatment of allergic diseases. The risk of asthma or AR development is increased in children whose parents, especially mothers, suffer from these disorders (19). Particular genes directly impact the immune response, both humoral and cellular. For instance, *IL-13+2044G/A, IL-4-590C/T* polymorphisms are associated with an increased risk of childhood asthma development (20).

Exposure to air pollutants is another important risk factor. It may explain the increased incidence of allergic diseases among children who live in industrial areas or whose relatives smoke (21). Pollutants (e.g. diesel emission particles) act as adjuvants and stimulate the production of cytokines that promote Th2 type response (22). Moreover, they cause oxidative stress that lowers corticosteroids responsiveness and reduces symptoms control (23).

It is presumed that immunomodulatory preparations that could possibly restore the natural balance in the immune response can conceivably be a game-changer in allergic diseases. BLs seem to be a good candidate for such treatment as they are characterized by a significant ability to modulate the immune response in a plethora of ways. What distinguishes them from conventional drugs used in the treatment of allergic diseases is that they can affect both innate and adaptive immune responses.

## Bacterial Lysates

### Methods of Preparation

Bacterial lysates are immunomodulatory preparations consisting of inactivated antigens derived from respiratory tract pathogens (24, 25). Bacterial species most commonly responsible for these infections are *Streptococcus pneumoniae, Haemophilus influenzae, Moraxella catarrhalis, Streptococcus pyogenes*, *Streptococcus viridans, Staphylococcus aureus, Klebsiella pneumoniae* and *Klebsiella ozaenae* (26).

The bacterial extracts are obtained in two ways, chemical or mechanical, which accordingly indicates different biological effects. Polyvalent chemical bacterial lysates (PCBLs) undergo alkaline lysis, during which the bacterial cell membrane is discomposed because of high pH in the range of 11.5-12.5 (27). Additionally, protein denaturation results in reduced immunogenicity after administration (28). Polyvalent mechanical bacterial lysates (PMBLs) endure lysis by either ultra-sonication or high pressure homogenization (27). This method is said to be more efficient as, within its course, the structures of bacterial antigens are not disrupted, which guarantees a better immune response (29).

### Basic Mechanisms of Action

Most of the evidence for the mechanism of action of BLs comes from animal and *in-vitro* studies. The mechanism of action presented by BLs is based upon a natural immune response provoked by pathogens, as it leads to stimulation of lymphoid tissue located in the mucosa, both locally and generally (30). Subsequently, through toll-like receptors (TLRs), BLs activate dendritic cells (DCs), being at the core of the innate and adaptive mucosal immunity (10, 31–33). As a result of that, proinflammatory cytokines are released, which consequently mobilize effector cells (27, 34, 35). The immunomodulatory effects of BLs reach not only cellular, but also humoral immunity (36). They promote antiviral cytokines production, macrophages, and NK cells activation in addiction to eosinophils mitigation (37, 38). Moreover, by their action, the Th2-associated immune response is weakened, with the effect of Th1/Th2 balance restoration (39–42).

### Application in Clinical Practice

The use of bacterial extracts implies significant immunomodulatory effects, and its safety has been confirmed through extensive research (43–45). For the past 100 years, the preparations have been successfully used to prevent recurrent respiratory tract infections (RTIs) (34, 36). Recent years have yielded information about the pivotal role of BLs’ ability to restore Th1/Th2 balance which allows prevention and treatment of various allergic diseases, such as asthma, AR, or AD (46–51). Studies confirming the effectiveness of BLs in the prevention and treatment of allergic diseases are discussed in more detail in paragraph: clinical effects of BLs therapy.

### Route and Regimens of Administration in Allergic Diseases

BLs can be administered orally, sublingually, and intranasally in a capsule, tablet, or spray form (29). The administration choice should be made considering each route’s slightly different metabolic and immunomodulatory effects (36). Nevertheless, the sublingual administration appears to be the most promising and safest alternative, as it provides robust and long-lasting immunity. This can possibly be attributed to the high concentration of dendritic cells in sublingual mucosa (52).

OM-85, which represents one of PCBLs, is advised to be taken orally with some fluid. It is available in two dosage forms. Capsules or sachets designated for children contain 3.5 mg of preparation, whereas adults should take 7 mg (53, 54). On the other hand, PMBLs, e.g. Ismigen is taken sublingually on an empty stomach. Irrespective of age it is administered in a 7 mg sublingual tablet (46). However, another PMBL, namely MV130 is a suspension prepared to be sprayed sublingually once per day (55). In asthma therapy, the intranasal spray form of BLs appears to be a promising option, which allows for a significant dose reduction compared to other routes of administration (56).

The treatment plan of using BLs in the therapy of allergic diseases is usually the same as that recommended in the prevention of RTIs. However, as the data suggest, it can be assumed that the period in which the therapy is started is essential in the case of seasonal allergic diseases. For instance, the treatment plan in seasonal AR (SAR) usually consists of prescribing one PMBL tablet sublingually per day for 10 days with 20 days of a break for three consecutive months, and the treatment should preferably be started a few days before the beginning of the pollen season (57). In a perennial AR (PAR), the dosing regimen is the same as described above; however, it is assumed that in this group of patients, repeating this regimen twice per year may provide additional benefits (58). **Table 1**. summarizes the BLs regimens for the prevention and treatment of allergic diseases used in clinical trials.

**Table 1:** Characteristics of included studies.

First author, year of publication (ref)	Subjects [n]	Mean age [years]	Intervention	Treatment regimen	Clinical and immunological outcomes BL compared to control
**Asthma treatment**					
Razi et al., 2010 (48)	75	2	OM-85 (PCBL) vs. placebo	1x/day for 10 days each month for 3 consecutive months	↓ RTIs, rate and duration of wheezing attacks
Lu et al., 2015 (37)	60	8.8	OM-85 (PCBL) vs. ICS	2 courses: 1x/day for 10 days each month for 3 consecutive months	↓ RTIs, frequency of asthma attacks and use of antibiotics ↑ serum NK, IL-10, IFN-γ/IL-4 ↓ serum IL-4
Han et al., 2016 (18)	136	2.2	OM-85 (PCBL) vs. ICS/aminophylline/antibiotics	1x/day for 10 days each month for 3 consecutive months	↓ frequency and duration of capillary bronchitis and asthma ↓ serum IL-4, IL-17 ↑ serum IL-10 and IFN-γ
Emeryk et al., 2018 (46) Bartkowiak-Emeryk et al., 2021 (57)	152	9.6	Ismigen (PMBL) vs. placebo	1x/day for 10 days each month for 3 consecutive months	↓ frequency and duration of asthma exacerbations, use of reliever medications ↑ time to the next asthma exacerbation ↑ serum T lymphocyte, CD4+CD25+FOXP3+, CD8+, CD3−CD16+CD56+ ↓ serum CD69+ and CD25+ subset of CD3+
Roßberg et al., 2020 (59)	606	5 weeks	Pro-Symbioflor vs. placebo	3x/day for 6 months	no influence on the development of asthma, AR, AD
Nieto et al., 2021 (60)	120	2	MV130 (PMBL) vs. placebo	1x/day for 6 months	↓ rate and duration of wheezing attacks, symptoms, and medication scores
**Allergic rhinitis treatment**					
Banche et al., 2007 (50)	41	29.3	Ismigen (PMBL) vs. placebo	1x/day for 10 days each month for 3 consecutive months	↓ symptom severity ↓ serum IL-4
Koatz et al., 2016 (61)	29	40.5	1^st^ year: standard optimized care 2^nd^ year: OM-85 (PCBL)	1x/day for 10 days each month for 3 consecutive months	↓ symptom severity, number of exacerbations ↑ serum and salivary secretory IgA
Meng et al., 2019 (11)	60	31.3	OM-85 (PCBL) vs. placebo	1x/day for 10 days each month for 3 consecutive months	↓ TNSS, itching score, nasal rhinorrhea score, sneezing score ↓ nasal IL-4, IL-13, eosinophils concentrations ↑ nasal INF-γ
Janeczek et al., 2021 (41)	70	9.2	Ismigen (PMBL) vs. placebo	1x/day for 10 days each month for 3 consecutive months	↓ TNSS, nVAS ↑ PNIF ↓ number of eosinophils in nasal swabs
**Atopic dermatitis treatment**					
Bodemer et al., 2017 (62)	170	2	OM-85 (PCBL) vs. placebo	1x/day for 9 months	↓ number of new flares

AD, atopic dermatitis; ICS, inhaled corticosteroid; nVAS, visual analogue scale for nasal symptoms; PCBL, polyvalent chemical bacterial lysate; PMBL, polyvalent mechanical bacterial lysate; PNIF, peak nasal inspiratory flow; RTIs, respiratory tract infections; TNSS, total nasal symptom score. ↓, decerase; ↑, increase.

## Innate Immune Response

**Figure 1:**
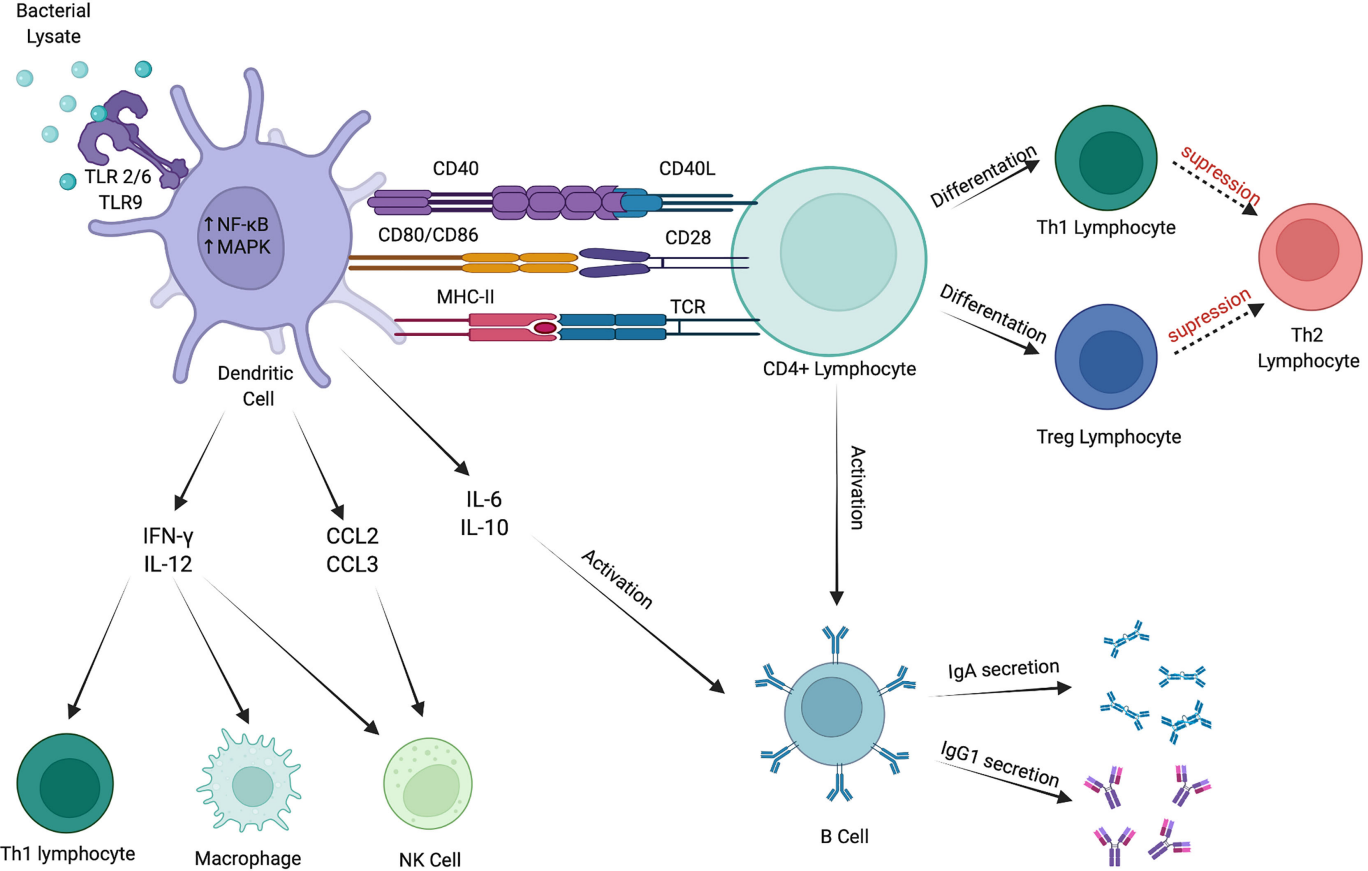
Immunomodulatory effects of bacterial lysates. BLs stimulate immune response *via* numerous pathways. They activate DCs *via* TLR2/6 and TLR9. Mature DCs produce cytokines that stimulate Th1 lymphocytes, macrophages and NK cells. Furthermore, they promote CD4+ lymphocytes to differentiate into Th1 and Treg subtypes and activate B cells to secrete IgA and IgG1. Created with BioRender.com.

The innate immune response is a first-line of host defense against harmful factors. It encompasses all tissues in the human body and involves hematopoietic as well as nonhematopoietic cells. These include DCs, neutrophils, eosinophils, macrophages, mast cells, and NK cells. Those cells express numerous receptors that can recognize pathogens and activate the immune response (**Figure 1**) (63).

### Toll-Like Receptors Activation

Toll-like receptors are dimeric proteins expressed on DCs and monocytes. They are essential for recognition and response to diverse microbial epitopes. Individual TLR reacts to a limited number of pathogen-associated molecular patterns (PAMPs). However, the whole TLR family (in humans, it comprises TLR1-TLR10) can recognize most of them (64). Identifying TLRs subtypes activated by BLs is an area of research.

Coviello et al. demonstrated that the immunomodulatory effect of BLs was dependent primarily on TLR4 signaling. In mice supplemented with BLs, the inflammatory cytokine production was promoted, and respiratory syncytial virus resistance was improved. Moreover, the significant role of TLR4 in B cell maturation and antibodies production was noted (65). However, not only TLR4 signaling pathway is crucial in DCs activation; TLR2 may also be involved in cytokine production induced by PCBL. Furthermore, NF-κB, one of the key transcription factors for macrophages and lymphocytes, was activated *via* TLR2 and TLR4 (32). On the other hand, Parola et al., in an *in-vitro* study, showed that PCBL does not activate the interferon regulatory factor (IRF) pathway, suggesting that TLR4 may not be involved in BLs recognition and signaling (31). Duggan et al. demonstrated that BLs in mice affected the innate lung response *via* TLR2/6 and TLR9 and improved resistance against infection. They concluded that the synergistic interaction of TLR2/6 and TLR9 supports the antimicrobial capacity of lung epithelium and may be the basis of new therapeutic approaches, both in inflammatory and allergic disorders (66). The majority of studies present mainly BLs’ impact on TLRs in non-allergic disorders; nevertheless, this knowledge may be helpful in allergy treatment.

### Dendritic Cells Activation and Maturation

Dendritic cells play a significant role in the immune system. They act as a bridge between innate and adaptive responses and comprise an essential subset of antigen-presenting cells necessary for antigen-specific proper T and B cell responses. Dendritic cells migrate to secondary lymphoid tissues, stimulating CD4+ T cells to differentiate into subtypes that depend on the nature of the activation signal and released cytokines (67, 68). Moreover, they can produce numerous mediators which activate B cells and promote antiviral response (69).

However, DCs need to maturate to achieve those abilities properly. Firstly, they have to be activated by binding a ligand by a specific TLR. Secondly, they migrate to lymph nodes and present the antigen to T cells and transmit activation signals *via* receptors on the surface of T cells: TCR (T cell receptor), CD28, and CD40L (70). Zelle-Rieser et al. suggest that BLs induce the terminal maturation of CD83+ DCs and their ability to stimulate T cells (71). Accordingly, other animal and *in-vitro* studies show a dose-dependent increase in the surface expression of MHC II, CD40, and CD86 on DCs populations due to the BLs supplementation (72, 73).

Numerous pathways lead mature DCs to affect innate immune response by producing antiviral cytokines. As mentioned before, BLs activate NF-κB and MAPK dependent pathways, which leads to IFN-α, IFN-β, and IL-8 production by DCs (31). Similarly, Dang et al. observed that PCBL can induce DCs to IFN-β production in a TLR adaptors Trif- and MyD88-dependent manner (74). In mice sensitized to ovalbumin and supplemented with BLs, enhanced IFN-γ production was observed (42). Similar findings were made by Bowman et al., who noted the increase in IFN-γ levels in rats who received orally OM-85 (39). Respectively, serum level of IFN-γ increased while IL-4 decreased in children with bronchiolitis treated with BLs. However, the research demonstrated decreased levels of NF-κB; thus, BLs’ impact on this molecule remains unexplained (75). Ruth et al. who examined the impact of two different BLs, consisting of different bacterial antigens, demonstrated in their *in-vitro* study that OM-85 induced the secretion of IFN-β. At the same time, Pulmonarom did not achieve similar results, which allows us to conclude that BLs’ composition significantly impacts the treatment outcome (76).

### Antiviral Cytokines Release

Activated DCs produce a plethora of cytokines that stimulate cells involved in the innate immune response. IFN-γ has a pleiotropic action in allergic disorders. As a mediator typical for type 1 of the innate immune response, it protects against intracellular pathogens through mononuclear phagocytes activation. Meng et al. demonstrated that the level of IFN-γ was substantially increased in the group of AR patients treated with PCBL (11). The meta-analysis also confirms this based on 19 studies evaluating bacterial lysate treatment in allergic patients, which showed a significant increase of IFN-γ after the supplementation with BLs (77). Lu et al. demonstrated similar effects; however, the authors concluded that the improvement in the asthma course was stimulated by the decrease of Th2-type cytokines rather than by the increase of IFN-γ (37). On the other hand, BLs can also reduce the levels of IFN-γ, which results in restricted mucosal inflammation. However, this phenomenon was observed in mice with experimental chronic rhinosinusitis; thus, comparing the results with the allergic ones may be troublesome (78).

BLs have some impact on the members of the IL-12 family. It consists of IL-12, IL-23, IL-27 and IL-35. IL-12 promotes Th1 differentiation and IFN-γ production, while IL-23 enhances Th17 response; IL-27 and IL-35 are mostly involved in regulating the number and function of T regulatory cells (both Treg and Tr1) (79). Byl et al. suggest that OM-85 involves the induction of IL-12 secretion by accessory cells (80). Similar findings were made by Lanzilli et al., who demonstrated a favorable impact of PMBL on IL-12 level in the *in-vitro* study (81). The aforementioned meta-analysis showed that the levels of IL-12 were increased after the treatment with BLs; however, only 2 out of 19 enrolled studies examined this phenomenon (77). On the other hand, in the *in-vitro* study by Parola et al., the expected positive impact of PCBL on IL-12 and IL-23 levels was not reached (31).

IL-10 is an important regulatory cytokine required to control allergic diseases. It can act directly on Th2 cells, regulating the survival of these cells and the severity of Th2-mediated allergic airway inflammation. Studies in asthmatic children have shown a significant increase in serum IL-10 concentration after BLs (18, 37).

Human beta-defensin-1 (hβD-1) is one of the essential antimicrobial peptides in epithelial tissues. It can promote DCs maturation *via* TLRs and inhibit the infectivity of multiple viruses (82). Liao et al. observed a significant increase in hβD-1 levels after PCBL supplementation in children with asthma and recurrent RTIs (83). Similar findings were made by Roth et al., who examined the impact of PCBL on bronchial epithelial cells infected with rhinovirus (84).

### Macrophages and NK Cells Activation

Macrophages are the most abundant immune cells that play a pivotal role in environmental allergen-induced airway inflammation in asthma and AR (85). What is important, they are a heterogenous population and divide into two subtypes: M1 macrophages induced by IFN-γ and M2 macrophages promoted by IL-4 and IL-13. Noticeably, they mirror the Th1/Th2 polarization; thus, distinguishing between activated subtypes is crucial while examining the impact of the BLs (86). Luan et al. demonstrated a positive impact of PCBL on murine macrophages activity *via* IFN-γ, TNF-α, IL-1β, and IL-6, which promoted the M1 subtype (32). PCBLs can act as a macrophage activator, promoting NO production and the translocation of NF-κB into the nucleus in murine bone marrow-derived macrophages (42).

NK cells are a subset of lymphocytes that principally participate in innate immunity by cytokine production, cytotoxicity, and activation of other cells. Notably, just like macrophages, they are divided into two subtypes that reflect the Th1/Th2 balance (87). IL-12 and IL-18 promoted by BLs stimulate NK1 polarization, which results in enhanced IFN-γ secretion and IgE production suppression (88). The study by Bartkowiak-Emeryk et al. showed an increase of NK cells in asthmatic children sublingually treated with PMBL (57). Moreover, DCs exposed to PCBL released increased levels of CCL2 and CCL3, chemokines important for chemoattraction of monocytes and NK cells (31). Migration of NK cells to inflamed tissue is vital for eliminating cells infected by viruses. This, in turn, leads to a decrease in the ratio of RTIs and thus to a reduction in the severity of the symptoms of allergic diseases.

## Adaptive Immune Response

**Figure 2:**
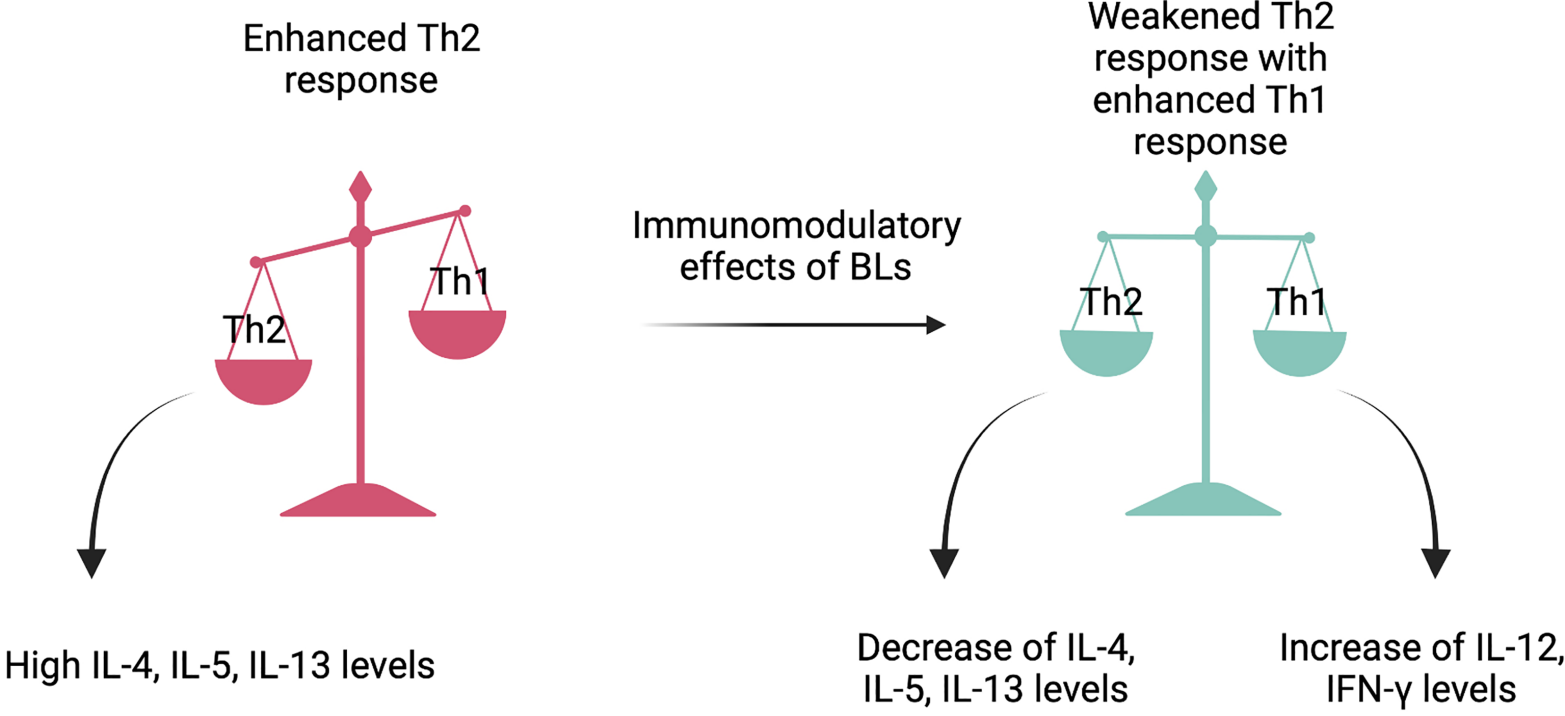
The Th1/Th2 balance restoration. Bacterial lysates act like immunomodulators and provide the maintenance of violated Th1/Th2 balance. By stimulating dendritic cells, they activate the production of numerous cytokines that weaken Th2 type response and enhance Th1 type response. The said restoration leads to allergy symptoms amelioration. Created with BioRender.com.

The adaptive immune response has a broader and more finely tuned repertoire of recognition for antigens than the innate response. It involves a particular interplay between antigen-presenting cells (APCs) and T and B lymphocytes resulting in effective pathogen-specific immunologic effector pathways. Moreover, it has a tremendous impact on the host immune homeostasis and immunologic memory (89). Such a variety of immune responses depends on the differentiation of naïve CD4+ T cells towards Th1, Th2, Th17, or Treg lymphocytes (90). **Figure 2** explains the immunomodulatory impact of BLs on restoring Th1/Th2 balance.

### Th1 Type Immune Response Promotion

Th1 cells promote the type-1 pathway known as ‘cellular immunity’ characterized by fighting against viruses and other intracellular pathogens, eliminating cancerous cells, and stimulating delayed-type hypersensitivity skin reactions (91). In response to an *in-situ* immunological stimulation, DCs present antigens to naïve lymphocytes and produce IL-12 to enhance their differentiation into specific Th1 cells (90). Furthermore, IFN-γ released by active NK cells, other CD4+, and CD8+ lymphocytes shows the same effect (92). Therefore, multiple animal studies and clinical trials examined the BLs’ impact on Th1 type immune response. In most of them, levels of IL-12 and IFN-γ were measured to conclude about said changes in Th1/Th2 balance. As mentioned in paragraph 4.3., BLs significantly increase the release of IFN-γ and IL-12; thus, their positive impact on Th1 type immune response remains valid (11, 41, 42, 77, 80, 81).

### Th2 Type Immune Response Inhibition

Th2 cells are lymphocytes whose high levels are specific for allergic disorders. They produce IL-3, IL-4, IL-5, IL-9, and IL-13 and stimulate type 2 immunity, characterized by high antibody titers and eosinophilia. This phenomenon is characteristic of AR in the upper respiratory tract, asthma in the lower respiratory tract, and atopic dermatitis in the skin; thus, restoring the said type of immunity acts to be an effective way of their treatment (93, 94). IL-4 was significantly downregulated in animal models after the treatment with BLs (39, 42, 95). Similar findings were made in patients with allergic disorders, in whom the decrease in IL-4 levels was accompanied by the improvements in the clinical courses of these diseases (11, 37, 50). Accordingly, IL-13 concentration was reduced in both animals and humans (11, 95, 96). IL-5 levels were measured only in murine models, and studies demonstrated that BLs cause their significant decreases (95, 96).

Both PMBL and PCBL seem to be effective in lowering eosinophil counts in blood and nasal and bronchoalveolar lavage fluids. Janeczek et al. investigated the PMBL’s impact on the clinical course of AR in children. They showed that sublingual administration of the drug resulted in decreased eosinophil count in nasal smears (41, 57). PCBL tested in adult patients and in murine models reduces eosinophil infiltration in nasal mucosa and lungs (11, 97). However, in the study by Rodrigues et al., PCBL did not reduce pulmonary eosinophilia; thus, it may not inhibit the development of asthma in a mice model. On the other hand, a significant reduction of IL-5 and IL-13 in bronchoalveolar lavage fluid was noted (96). Bearing in mind that most research proved that BLs reduce levels of IL-4, IL-5, IL-13, and eosinophils, we can presume they have a positive impact on Th2 type response decrease.

### Tregs Activation

Regulatory T cells are subsets of CD4+ T cells that modulate the immune response, maintain tolerance to self-antigens, and prevent autoimmune diseases. They stem from CD4+ T cells which acquired CD25 and FOXP3 molecules because of weak self-antigen specificity (98). In a murine asthma model, oral administration of PCBL induced an increase in the number of CD4+CD25+FOXP3+ lymphocytes in intestinal lamina propria and respiratory tract mucosa (99). Similar findings showed a study that examined PMBL’s impact on Treg cells. CD4+CD25+FOXP3+ lymphocytes significantly increased in asthmatic children treated sublingually with Ismigen (57). The mechanism of Treg cells’ action is complicated and multidirectional. Among others, they suppress the proliferation and maturation of Th2 cells; thus, their activation seems to be effective in allergy treatment.

### B Cells Activation

B cells, along with T cells, are two major cell subsets involved in adaptive immunity. Once activated, B cells maturate into antibody-secreting plasma cells. Initially, plasma cells produce IgM class antibodies, but eventually, they switch to IgA, IgG, or IgE. This process in part depends on the cytokine milieu e.g. IL-4 and IL-13 promote the switch to IgE, while IL-10 and IFN-γ promote IgA and IgG1. As mentioned before, allergic disorders are characterized by high IgE levels; thus, the direction in which Ig-switch goes may lead either to allergy amelioration or worsening (100). BLs seem to improve the immunoglobulin profile in asthma and AR. Huber et al. demonstrated a decrease of both total and asIgE in mice orally treated with PCBL (42). Furthermore, in rat model of food allergy, animals treated with BLs presented a significant decrease of asIgE (101). On the other hand, asIgE levels did not decrease after the PMBL therapy in children with SAR. However, in a placebo group, a significant increase was observed; thus, the positive impact of the mixture was achieved (41). IgA protects against microbes on epithelial barriers as the main immunoglobulin associated with mucosal membranes. Koatz et al. demonstrated a significant increase in serum and salivary secretory IgA in patients with asthma, AR, and chronic obstructive pulmonary disease after PCBL supplementation (61). Furthermore, BLs seem to positively impact IgA levels in children with bronchiolitis and recurrent RTIs associated with immunoglobulins deficiency (75, 102). Similarly, IgG1 levels rise after the BLs supplementation, even in patients with IgG deficiencies, and enable them to reach the average age levels of these immunoglobulins (103).

## The Route of Administration of BLs and Immunological Effects

As described before, BLs can be administered in three ways, each having specific implications on the immune system.

BLs can be administered orally (53). It is claimed that this route of administration promotes a reorganization of the gut and lung microbiota by maintaining immune homeostasis through the gut-lung axis (104). The process starts with bacterial extracts sampled by microfold cells, followed by gastrointestinal absorption to Peyer patches that are part of gut lymphoid tissue. This leads to antigen presentation to the mucosal DCs (105). Consequently, the antigen-specific T and B lymphocytes are stimulated; this process is partly modulated by cytokines: TGF-β and members of the tumor necrosis factor family (34, 106). In the end, B-cell isotype switching to IgA occurs (107). Because of described processes, the flow of the immune cells begins (32, 108). Activated DCs, lymphocytes, and lymphoblasts begin their migration to the mesenteric lymph nodes, through which they enter the blood system to eventually locate in distant mucosa lymphoid tissue (107). This migration enhances the defense against pathogens by stimulating pathogen cells and increasing not only IgA concentrations, but also IgA-producing plasma cells (53, 109). Thus, BLs administered orally play a pivotal role in the mucosal immune response.

Plasma cells can be found within nasal tissues; they secrete antibodies, especially IgA (110). What is more, they can produce antimicrobial peptides (AMPs), which protect the airway epithelium from colonization (111). BLs administered intranasally are found to induce the production of one specific AMP, hβD-2. The process is linked to the stimulation of TLRs, which interact with intranasal macrophages and DCs by the bacterial molecule compounds of lysates (112, 113). Moreover, there is evidence that stimulation of intranasal tissues in mice by BLs leads to interaction with several pattern recognition receptors (PRRs) and engagagement of effector cells directly on the epithelial surfaces, which plays a role as a ‘pre-alert scenario’ that prevents further respiratory infection and allows for immediate elimination of pathogens (106, 114). Animal studies also show that airway administration of BLs can cause an acute inflammatory response that lasts a minimum of 7 days (115).

It is believed that the sublingual route of BLs administration, in particular those obtained with mechanical lysis, may result in better clinical efficacy. It has been provided by a study by La Mantia et al. on a group of 120 children with nasopharyngitis or otitis media. Researchers demonstrated greater protective efficacy of sublingual PMBL than oral PCBL (116). This route of delivery is associated with systemic Th1 responses, increases in IgG with decreases in IgE concentrations, as well as attenuation of eosinophil recruitment (10, 81). It is emphasized that the sublingual administration provides a specific-humoral and cell-mediated immunity that lasts up to 4 months after the last immunization. A strong and sustained immune response to pathogens is possible thanks to generation and stimulation antigen-specific memory CD4+ and CD8+ cells (117, 118). Moreover, due to the high concentration of DCs observed in sublingual mucosa, PAMPs can be presented and recognized by PRRs more effectively (119). Studies confirm that mucosal DCs interact with lipopeptide antigens more eagerly than macrophages; thus, enabling an innovative noninvasive vaccine option not requiring any additional adjuvants (120). Furthermore, smaller doses of BLs are necessary when administered sublingually, hence the absence of medicament’s neutralization with gastric acidity (121). One encouraging benefit is excellent compliance with sublingual bacterial lysate therapy in all children, even the youngest (122).

## Clinical Effects of BLs Therapy

Numerous studies proved BLs’ efficacy in treating not only recurrent RTIs but also various allergic diseases (77). As aforementioned, thanks to their ability to induce both humoral and cellular immunity, bacterial preparations restore the immune imbalance at the core of allergic pathogenesis (18). Here we describe and analyze the impact of BLs on the prevention and treatment of asthma, AR, and AD with particular consideration of pediatric patients (**Table 1**).

### Asthma

Razi and colleagues assessed the effect of PCBL in preventing wheezing attacks induced by acute respiratory illnesses in 75 preschool children with recurrent wheezing. Patients were treated for 3 consecutive months with OM-85 or a placebo following the typical regimen. Then they were followed up for 9 months. It has been shown that OM-85 significantly reduced the rate and duration of wheezing attacks. In addition, this study also reported a reduction of RTIs, which was considered to be most likely to explain the observed reduction in wheezing attacks (48).

In 2015 Lu et al. presented the results of a study that they conducted in order to assess the effect of OM-85 combined with conventional treatment on the course of asthma in children. Sixty patients were included in the study and divided into two groups, one treated with PCBL in combination with inhaled corticosteroid (ICS), the second only with ICS. In the PCBL-treated group, there was a significant reduction of RTIs, the frequency of asthma attacks, and antibiotic therapy (37).

Another study was conducted by Han et al., who focused on the effects of BLs therapy on capillary bronchitis secondary bronchial asthma. They enrolled 136 children who were divided into two groups, the control one (n=62) treated with ICS, aminophylline, and antibiotics, and the observation one (n=74) additionally receiving OM-85 orally. The follow-up period was set out for 12 months. The results in the PCBL group presented a significant decline in the frequency and duration of capillary bronchitis and asthma compared to the control group (18).

A randomized, double-blind, placebo-controlled study conducted by Emeryk et al. enrolled 152 children divided into the placebo group and the observation group undergoing PMBL therapy. The trial lasted 9 months, and its’ results indicated a significant reduction in the frequency and duration of asthma exacerbations and in the use of reliever medications in the PMBL group vs. placebo group. This intervention also prolonged the time to the next exacerbation (46). Laboratory results from this study were presented in 2021 by Bartkowiak-Emeryk et al. (**Table 1**) (57).

Roßberg et al. conducted a randomized, placebo-controlled study to evaluate the effect of oral BLs administered in infancy on the risk of developing AD, AR, and asthma. The study enrolled 606 infants with a family history of allergic diseases, 402 of which were followed until reaching the school age of 6-11 years. The observation group was treated with orally applied BLs three times daily from 5 weeks to 7 months of life, with the placebo group adequately following the same regimen. BLs have not been shown to reduce the risk of allergic diseases, for instance asthma was diagnosed in 9% of patients in the observation group and in 6.6% of patients in the placebo group (59).

In 2021 Nieto et al. presented the results of a randomized, double-blind, placebo-controlled study in which they assessed the efficacy and safety of MV130 in preventing wheezing attacks in children. However, it should be mentioned that patients with allergies were excluded from this trial. The whole study lasted 12 months, with 6 months of treatment plus 6 additional months of follow-up. There was a significant reduction in the number of wheezing attacks in the observation group, and a reduction in symptoms and medication scores compared to the placebo group. Moreover, a high safety profile of this therapy has been demonstrated (60).

Finally, the ORBEX study, a large, multicenter, randomized, double-blind, placebo-controlled study, is currently ongoing in the USA. The study enrolls children aged 6-18 months presenting a high risk of asthma development (diagnosed with AD and/or with a family history of asthma). The main objective of this study is to assess whether OM-85 given for 10 days per month for two consecutive years can extend the time to the first episode of wheezing or lower respiratory tract illness during a three-year observation period without therapy. According to data available on clinicaltrials.gov, the study is expected to end in 2025 (123).

Literature data indicate that therapy with BLs does not have a preventive effect on the occurrence of asthma, but it reduces the frequency of exacerbations in patients already diagnosed with asthma.

### Allergic Rhinitis

In 2007 Banche et al. conducted a trial to assess the PMBLs’ influence on the clinical manifestations of AR. 41 patients with SAR and PAR were enrolled and randomly assigned to the observation group (n=26) and the placebo group (n=15). The participants were administered either placebo or PMBL for 10 days each month for 3 consecutive months, with no other antiallergic drugs. Eventually, the results showed significant alleviation of symptoms in the observation group with the decrease of nasal congestion, rhinorrhea, and ocular symptoms (50).

To further expand the research, Meng et al. evaluated the clinical effects of OM-85 in 60 patients with PAR. The participants were randomly assigned to the PCBL group (n=30) and the placebo group (n=30), with an average age of 33 and 29 years, respectively. Both groups followed the regimen of oral administration in 3 cycles consisting of consecutive 10 days of intake followed by a 20-days break. Clinical symptoms were measured at baseline, immediately after the third cycle of PCBL, and 4 and 8 weeks after the end of treatment. In the end, the PCBL group presented a significant decrease in medication score, a drop in the total nasal symptom scores, itching score, nasal rhinorrhoea score, and sneezing score (11).

In 2021 Janeczek et al. presented the results of a double-blind, placebo-controlled study investigating the impact of PMBL on children with grass pollen-induced AR. The study included 70 patients aged 5-17 years who were randomly assigned to the observation and placebo groups. The numerous measured parameters included the total nasal symptom score, total ocular symptom score, visual analogue scale, and peak nasal inspiratory flow. The observation group received a PMBL tablet sublingually once per day in the fasting state on the first 10 days of each month for 3 months. Consistently, the placebo group followed the same regimen. Finally, the PMBL group showed a significant decrease in total nasal symptom score, visual analogue scale for nasal symptoms, as well as a significant increase in peak nasal inspiratory flow compared with the placebo group (41).

What is more, Koatz et al. presented an open-label, sequential study concerning the implications of OM-85 on RTIs in patients with AR, asthma, or chronic obstructive pulmonary disease. The included 84 patients were between the ages of 16-65 years and were administered OM-85 orally for 10 days per month, for 3 months, with a 6-month follow-up. Before the study, patients received only standardized optimized care for one year. In the end, the observation group showed a decrease in the frequency of RTIs and primary disease exacerbation rates (61).

Based on the above-mentioned studies, BLs appear to be a promising therapeutic option for AR patients, including those with SAR and PAR.

### Atopic Dermatitis

As mentioned before, the use of oral BLs in early infancy over 6 months does not affect the development of AD at school age (59).

In 2017 Bodemer et al. presented the results of the study concerning the clinical efficacy of OM-85 in the treatment of children with AD. One hundred seventy patients were enrolled in the study. Apart from conventional therapy with emollients and topical corticosteroids, the participants took one capsule of OM-85 or a placebo daily for 9 months. The results confirmed BLs efficacy, as patients in the observation group had significantly fewer and delayed new flares (62).

We have to conclude that there is an urgent need for high-quality and large sample size studies on the clinical efficacy of BLs with different bacterial antigen compositions, methods of preparation, and routes of administration.

## Conclusions

As common as allergic disorders are, there is yet to know about their exact pathogenesis and indications. With an escalating prevalence, it is crucial to thoroughly clarify the individual predispositions and risk factors for atopic diseases development. Although various burdensome clinical manifestations characterize the heterogeneous group of allergies, the search for an effective, inclusive, and noninvasive treatment continues to last.

BLs promise new therapeutic possibilities in AD, AR, and asthma; however, their exact effects are not fully elucidated. Various clinical effects are observed with the main differences in PCBLs and PMBLs’ obtainment. As stated before, PCBLs present reduced immunogenicity due to protein denaturation. PMBLs, on the other hand, are known to induce a better immune response. Further differences among preparations are caused by the diversity of bacteria species used to obtain the mixture. Little is currently known about the exact impact of specific antigens on the immune response; thus, further research is still needed.

Our paper shows a significant indissolubility between innate and adaptive responses. BLs act as immunomodulators and promote DCs’ activation and maturation *via* TLR 2/6 and TLR 9. Stimulated DCs produce multiple cytokines that affect innate and adaptive responses; thus, they appear to be a bridge between them. Indeed, the enhanced innate immune response plays a vital role as the first-line defense against pathogenic agents, which allows the host to tackle atopic reactions and maintain homeostasis. IFN-γ and IL-12 deserve special attention, as they promote macrophage and NK cell development along with Th1 and Treg lymphocytes differentiation. Furthermore, B cells activation and IgA and IgG1 secretion provoked by IL-6 and IL-10 lead to allergy symptoms reduction. Finally, Th2 lymphocyte diminution is a significant sign of allergy restriction.

On the other hand, the mentioned immunological outcomes may vary according to the way of BLs administration. That being said, the sublingual route of administration, in particular the preparations obtained by the mechanical lysis, seems to be the most effective; however, this hypothesis is not entirely proven. Furthermore, the other medications’ influence on BLs action is still under research, as the examined interactions regard mostly antiallergic preparations. The results of the studies indicate a beneficial effect of BLs therapy on the clinical course of allergic diseases. Furthermore, it is extremely important to identify those patients who may significantly benefit from this therapy. Notwithstanding, the exact number of high-quality RCTs remains insufficient. Furthermore, they vary in study design and patients’ clinical picture; thus, comparing their findings is troublesome.

To sum up, BLs are promising immunomodulators that may change therapeutic approaches to allergic diseases. Nevertheless, more rigorous and detailed trials are demanded to draw definitive conclusions about the efficacy of BLs in terms of atopic diseases’ prevention and treatment.

## Author Contributions

AK contributed to the conception and design of the work, acquired, and analyzed of references for the work, wrote the first draft the manuscript; MK contributed to the conception and design of the work, acquired, and analyzed of references for the work; wrote the first draft the manuscript; KJ contributed to the conception and design of the work;, acquired, and analyzed of references for the work, drafting the work or revising it critically for important intellectual content; MZ contributed to the conception and design of the work;, acquired, and analyzed of references for the work, drafting the work or revising it critically for important intellectual content; AE contributed to the conception and design of the work;, acquired, and analyzed of references for the work, drafting the work or revising it critically for important intellectual content. All authors contributed to the article and approved the submitted version.

## Funding

The authors declare that this study received funding from Celon Pharma. The funder was not involved in the study design, collection, analysis, interpretation of data, the writing of this article or the decision to submit it for publication.

## Conflict of Interest

The authors declare that the research was conducted in the absence of any commercial or financial relationships that could be construed as a potential conflict of interest.

## Publisher’s Note

All claims expressed in this article are solely those of the authors and do not necessarily represent those of their affiliated organizations, or those of the publisher, the editors and the reviewers. Any product that may be evaluated in this article, or claim that may be made by its manufacturer, is not guaranteed or endorsed by the publisher.
